# Coordination of IFT20 With Other IFT Components Is Required for Ciliogenesis

**DOI:** 10.1002/jcla.70000

**Published:** 2025-04-07

**Authors:** Weishu Wang, Ying Shan, Ruming Liu, Dengwen Li, Jun Zhou, Quanlong Lu, Huijie Zhao

**Affiliations:** ^1^ Department of Genetics and Cell Biology, State Key Laboratory of Medicinal Chemical Biology, Haihe Laboratory of Cell Ecosystem, College of Life Sciences Nankai University Tianjin China; ^2^ Center for Cell Structure and Function, Shandong Provincial Key Laboratory of Animal Resistance Biology, College of Life Sciences Shandong Normal University Jinan China

**Keywords:** axoneme, basal body, ciliogenesis, cilium, IFT20

## Abstract

**Background:**

Primary cilia are organelles formed on the cell surface. They can act as cellular antennae to sense signals and play important roles in various biological processes. Abnormalities in primary cilia lead to a variety of diseases collectively known as ciliopathies. Intraflagellar transport protein 20 (IFT20) has been implicated in ciliogenesis.

**Methods:**

*IFT20* knockout cell lines were established using the CRISPR‐Cas9 gene editing technology. The GFP‐IFT20 plasmid was constructed with the Gateway cloning system. Protein levels were detected via immunoblotting, and the localization of IFT20, acetylated α‐tubulin, ARL13B, CP110, MKS3, IFT88, and IFT140 in wild‐type and *IFT20* knockout cells was examined by immunofluorescence microscopy. The fluorescence intensities were analyzed using ImageJ. Data quantifications and mass spectrometry results were analyzed using GraphPad Prism and Metascape.

**Results:**

The IFT20 deficiency impaired ciliogenesis and reduced cilium length. IFT20 depletion did not affect the removal of centriolar coiled‐coil protein 110 (CP110) from the mother centriole or the recruitment of Meckel–Gruber syndrome type 3 (MKS3) to the transition zone. Mass spectrometry analysis revealed that proteins interacting with IFT20 were mainly IFT components. *IFT20* knockout decreased the levels of both IFT88 and IFT140, and abrogated IFT88 localization at the basal body and ciliary axoneme. *IFT20* knockout also impaired IFT140 localization at the ciliary axoneme but did not affect its localization at the basal body.

**Conclusions:**

IFT20 is involved in ciliogenesis by regulating the level and localization of other IFT proteins and may have important implications in ciliopathies and related diseases.

## Graphical abstract

Intraflagellar transport is a bidirectional transport system essential for ciliogenesis. Using the *IFT20* knockout cell lines established in this study, we found that IFT20 deficiency significantly impaired ciliogenesis and reduced cilium length. Further investigation revealed that IFT20 interacted with other IFT proteins and regulated their levels and localization to function in ciliogenesis.

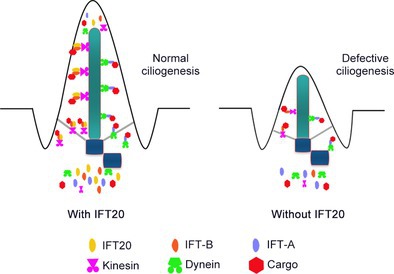

## 1 Introduction

Cilia are microtubule‐based organelles protruding from the cell surface with important functions. In vertebrates, cilia are present on the surface of various types of cells [1, 2, 3]. Based on their structures and functions, cilia can be divided into primary cilia and motile cilia [4]. Primary cilia are the most widely distributed and function as cellular antennae, responsible for sensing extracellular mechanical, chemical, and biological signals, and transmitting these signals to the cell, playing an indispensable role in cell proliferation and differentiation during embryonic development [5, 6, 7]. Abnormalities in the structure or function of cilia lead to diseases such as blindness, hydrocephalus, congenital heart disease, polycystic kidneys, and mental retardation, collectively known as ciliopathies [7, 8, 9, 10, 11, 12, 13, 14, 15]. The main structures of primary cilia include the basal body, ciliary axoneme, and ciliary membrane. The basal body is derived from a mature centriole and consists of nine triplet microtubules. The ciliary axoneme is assembled from the basal body and comprises nine doublet microtubules. The outermost layer of the cilium is the ciliary membrane, which is connected to the cell membrane at a base called the ciliary pocket [16, 17, 18, 19, 20].

Intraflagellar transport (IFT) is a bidirectional transport system in cilia that comprises motor proteins (kinesin 2 and cytoplasmic dynein 2) and IFT complexes [21, 22, 23, 24]. Anterograde transport of particles from the base to the tip of the cilium is responsible for transporting substances required for cilium assembly, while retrograde transport serves to recycle IFT particles from the tip to the base of the cilium. IFT complexes are organized into two subcomplexes, IFT‐A and IFT‐B. The IFT‐A complex contains 6 proteins, and the IFT‐B complex contains 16 proteins [25, 26]. The smallest of the IFT proteins is IFT20 [27], the only IFT member that localizes to the basal body, ciliary axoneme, and Golgi apparatus. IFT20 has many extraciliary functions, such as transporting T cell receptors and linkers to the immune synapse to activate T cells [28, 29] and interacting with dynein during vesicular transport from late endosomes to the trans‐Golgi network [30]. IFT20 is also involved in targeting vesicular transport to the postsynaptic dendritic terminals of neurons [31] and regulates the recycling of β1‐integrin in epidermal cells toward the focal adhesion [32]. In addition, deletion of IFT20 in the mouse kidney leads to misorientation of the mitotic spindle and cystic kidney disease [33].

IFT20 is also required for male fertility and spermiogenesis in mice through its function in transporting cargo proteins for sperm flagellum formation [34]. Interestingly, deletion of IFT20 in mouse retinal pigment epithelium leads to retinal degeneration [35], which coincides with the transport of the ciliary membrane protein, polycystin‐2, or dynein, from the Golgi apparatus to the cilium [27, 33, 36, 37]. However, the molecular mechanism of how IFT20 participates in ciliogenesis remains poorly understood. In this study, we established *IFT20* knockout cell lines and found that the level and localization of IFT proteins were affected to different extents by IFT20 deletion. These findings provide novel insights into the function of IFT20 in ciliogenesis and suggest an exquisite interplay between IFT complex proteins.

## 2 Material and Methods

### 2.1 Cell Culture

Human retinal pigment epithelial (RPE1) cells were obtained from ATCC and cultured in DMEM/F12 medium containing 10% fetal bovine serum, penicillin, and streptomycin. Human embryonic kidney epithelial (HEK293T) cells were obtained from ATCC and cultured in DMEM medium supplemented with 10% FBS, penicillin, and streptomycin. All cell lines were cultured at 37°C with 5% CO_2_. To induce cilium formation, cells were cultured in a serum‐free medium for 24 h.

### 2.2 Plasmid Construction

Full‐length human *IFT20* (NM_001267774) and *IFT88* (NM_001353567) were obtained from the DNASU Plasmid Repository (Arizona State University), PCR‐amplified, and subcloned into the recombinational donor vector pDONR221 to generate entry clones (ThermoFisher). LR recombination reactions between entry clones and desired gateway destination vectors (Kit #1000000107, Addgene) were performed to generate the expression constructs.

### 2.3 Establishment of *IFT20* Knockout Cell Lines

RPE1 cells were transfected with plasmids containing Cas9 and guide RNAs targeting the exon3 region of *IFT20*. Colonies were derived from single cells, and two clones were selected by sequencing. The single‐guide RNA (sgRNA) was designed by using Benchling. The sequences of sgRNA and primers were as follows: sgRNA(5′‐AGTGTAGCCCTGCTTCACCC‐3′), *IFT20*‐F(5′‐GCCTGTGACAAGCAAGCGGA‐3′), and *IFT20*‐R(5′‐GTGTTTGGTTTGACTGGTGC‐3′).

### 2.4 Antibodies

The following primary antibodies were used: rabbit anti‐IFT20 (Proteintech, 13,615‐1‐AP; IB: 1:800; IF: 1:200), rabbit anti‐CP110 (Proteintech, 12780‐1‐AP; IF: 1:1000), mouse anti‐acetylated α‐tubulin (Sigma‐Aldrich, T6793; IF: 1:10000), rabbit anti‐CEP164 (Proteintech, 22227‐1‐AP; IF: 1:1000), rabbit anti‐ARL13B (Proteintech, 17711‐1‐AP; IF: 1:1000), chicken anti‐CEP164 (Our lab; IF: 1:500), rabbit anti‐GM130 (Proteintech, 66662‐1‐Ig; IF: 1:1000), rabbit anti‐MKS3 (Bicell Scientific, 90103; IF: 1:200), mouse anti‐GFP (Roche, 11814460001; IB: 1:1000), rabbit anti‐IFT88 (Proteintech, 13967‐1‐AP; IB: 1:5000; IF: 1:200), rabbit anti‐IFT140 (Proteintech, 17460‐1‐AP; IB: 1:5000; IF: 1:1000). Alexa Fluor 488‐, 568‐ or 647‐conjugated secondary antibodies were purchased from Life Technologies.

### 2.5 Immunoblotting

Samples were lysed in a lysis buffer (Solarbio) with PMSF (Solarbio). Samples were separated by SDS–polyacrylamide gel electrophoresis. Proteins were then transferred onto polyvinylidene difluoride membranes. The membranes were blocked in 5% skimmed milk at room temperature for 2 h and incubated with primary antibodies overnight at 4°C, followed by secondary antibodies for 1 h at room temperature. Protein bands were visualized by using the luminol reagent (Millipore).

### 2.6 Immunofluorescence Staining

Cells grown on glass coverslips were fixed in freshly prepared 4% paraformaldehyde (PFA) for 10–20 min at room temperature or in ice‐cold methanol for 3 min and permeabilized in BSA solution (1% BSA, 0.1% Triton X‐100 in PBS) for 10 min at room temperature. Cells were incubated with primary antibodies at 4°C overnight and then with secondary antibodies for 1 h at room temperature. Finally, cells were fixed to the slide with a DAPI‐containing sealer and examined with a Zeiss LSM710 confocal microscope.

### 2.7 Immunoprecipitation

Samples were lysed in a lysis buffer (10 mM Tris, 150 mM NaCl, 6 mM EDTA, 1 mM EGTA, 2%Triton X‐100, 20% glycerol, and 2 mM sodium pyrophosphate, pH 7.5) with a protease inhibitor cocktail (EpiZyme) at 4°C for 0.5 h. Samples were centrifuged at 4°C, 12,000 rpm for 20 min. One hundred microliters of the supernatant were taken as lysate, and the remaining samples were incubated with anti‐GFP beads (Abcam) at 4°C overnight. Beads were then washed with the lysis buffer several times. Extracted proteins with beads were examined by immunoblotting.

### 2.8 Statistical Analysis

All the data in this study are presented as mean ± SEM. Student's *t*‐test was used to compare the difference between the two groups. For multiple‐condition comparisons, one‐way ANOVA was performed. The data were considered significant when *p* < 0.05. All the experiments were repeated at least three times. Bioinformatics analysis was generated by Metascape (https://metascape.org).

## 3 Results

### 3.1 Establishment of *IFT20* Knockout Cell Lines

**FIGURE 1:**
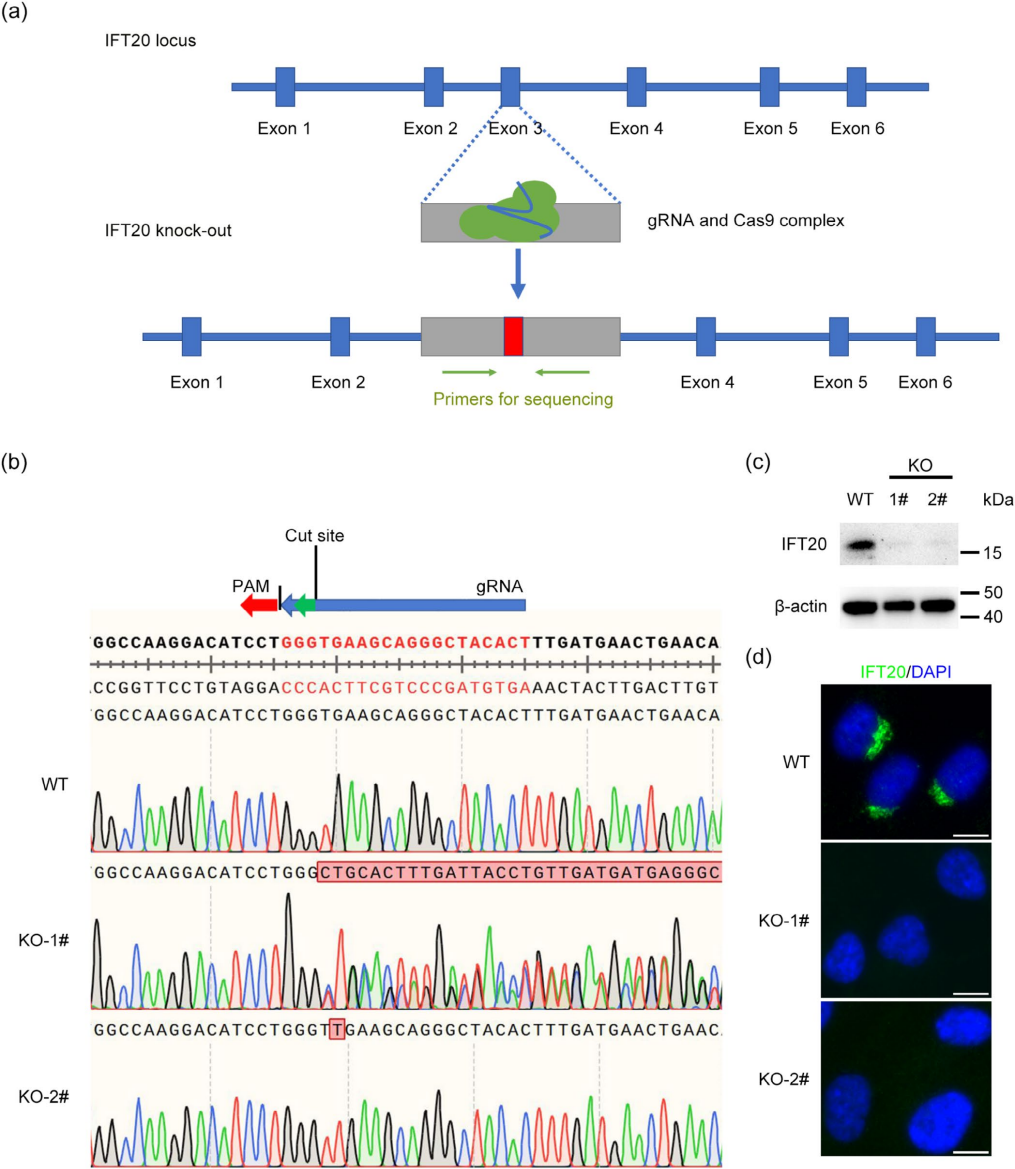
Establishment of *IFT20* knockout cell lines. (a) A scheme of the strategy used for constructing the *IFT20* knockout cell line. (b) Comparison of the *IFT20* sequences of wild‐type and *IFT20* knockout cells by SnapGene. (c) Detection of IFT20 depletion efficiency by immunoblotting. (d) Analysis of IFT20 expression by immunofluorescence staining (IFT20, green; DAPI, blue. Scale bar, 10 μm).

To explore the role of IFT20 in ciliogenesis, we sought to establish stable *IFT20* knockout cell lines using the CRISPR‐Cas9 gene editing technology. The core component of this technology is Cas9, which is an RNA‐directed DNA endonuclease. Cas9 can precisely recognize, bind, and cleave target DNA sequences according to the guidance of the sgRNA it carries, enabling the editing, addition, or deletion of genomes. We used the online tool Benchling to design the sgRNA of *IFT20* with relatively high scores and selected one sgRNA targeting the third exon of *IFT20* (Figure 1a). The vector plasmids were digested and ligated with the annealed products of the sgRNA and verified by sequencing with specific primers. HEK293T cells were transfected with a plasmid encoding Cas9 and sgRNAs targeting *IFT20*, a lentiviral packing plasmid (pSPAX2), and an envelope plasmid (pMD2.G) to produce viruses. After transfection for 48 h, the supernatant was collected and used to infect RPE1 cells. After drug screening, two clones with different splicing patterns were selected by sequencing (Figure 1b). The efficiency of IFT20 depletion in these two clones was then verified by immunoblotting (Figure 1c). Immunofluorescence microscopy revealed that IFT20 was localized to the Golgi apparatus in wild‐type RPE1 cells, whereas this localization disappeared in the *IFT20* knockout cell lines (Figure 1d). These results demonstrate the successful establishment of *IFT20* knockout cell lines.

### 3.2 IFT20 Is Indispensable for the Formation of Primary Cilia

**FIGURE 2:**
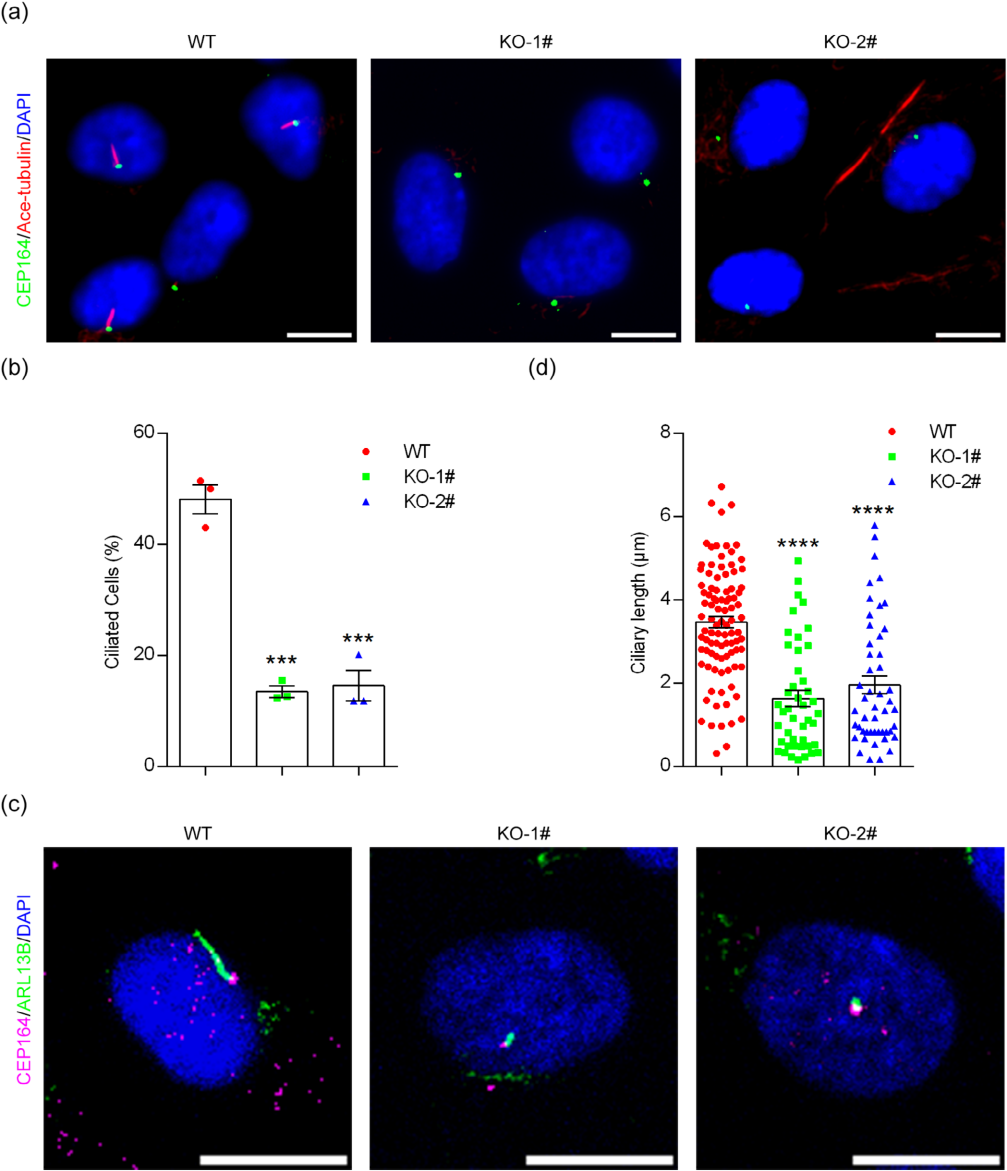
IFT20 is indispensable for the formation of primary cilia. (a) Immunofluorescence microscopy to detect the percentage of ciliated cells in wild‐type and *IFT20* knockout cell lines following serum starvation (CEP164, green; Ace‐tubulin, red; DAPI, blue. Scale bar, 10 μm). (b) Quantification of the percentage of ciliated cells following serum starvation (*n* = 3 independent experiments). (c) Immunofluorescence microscopy of ARL13B in wild‐type and *IFT20* knockout cell lines following serum starvation (CEP164, magenta; ARL13B, green; DAPI, blue. Scale bar, 10 μm). (d) Quantification of the ciliary length following serum starvation (*n* = 100 cilia from three independent experiments). Data are presented as mean ± SEM. ***, *p* < 0.001; ****, *p* < 0.0001.

Primary cilia play essential roles in diverse physiological processes, and dysregulation of ciliary proteins, including IFT20, leads to abnormalities in various organs. To confirm the requirement of IFT20 for ciliogenesis, we examined the effect of IFT20 depletion on the status of primary cilia. Immunofluorescence microscopy was used to investigate the percentage of ciliated cells following serum starvation, which is widely used to induce ciliogenesis. Using antibodies against acetylated α‐tubulin (Ace‐tubulin, a marker of the ciliary axoneme) and centrosomal protein 164 (CEP164, a marker of the basal body), we found that depletion of IFT20 resulted in fewer cilia (Figure 2a,b). In addition, we examined whether *IFT20* knockout affects ciliary length. We labeled cilia with an antibody against ADP‐ribosylation factor‐like 13B (ARL13B, a marker of the ciliary membrane). We found that the length of cilia was significantly shortened in *IFT20* knockout cells compared to that in wild‐type cells (Figure 2c,d). Collectively, these results reveal a critical role for IFT20 in ciliogenesis.

### 3.3 IFT20 Is Not Required for the Early Steps of Ciliogenesis

**FIGURE 3:**
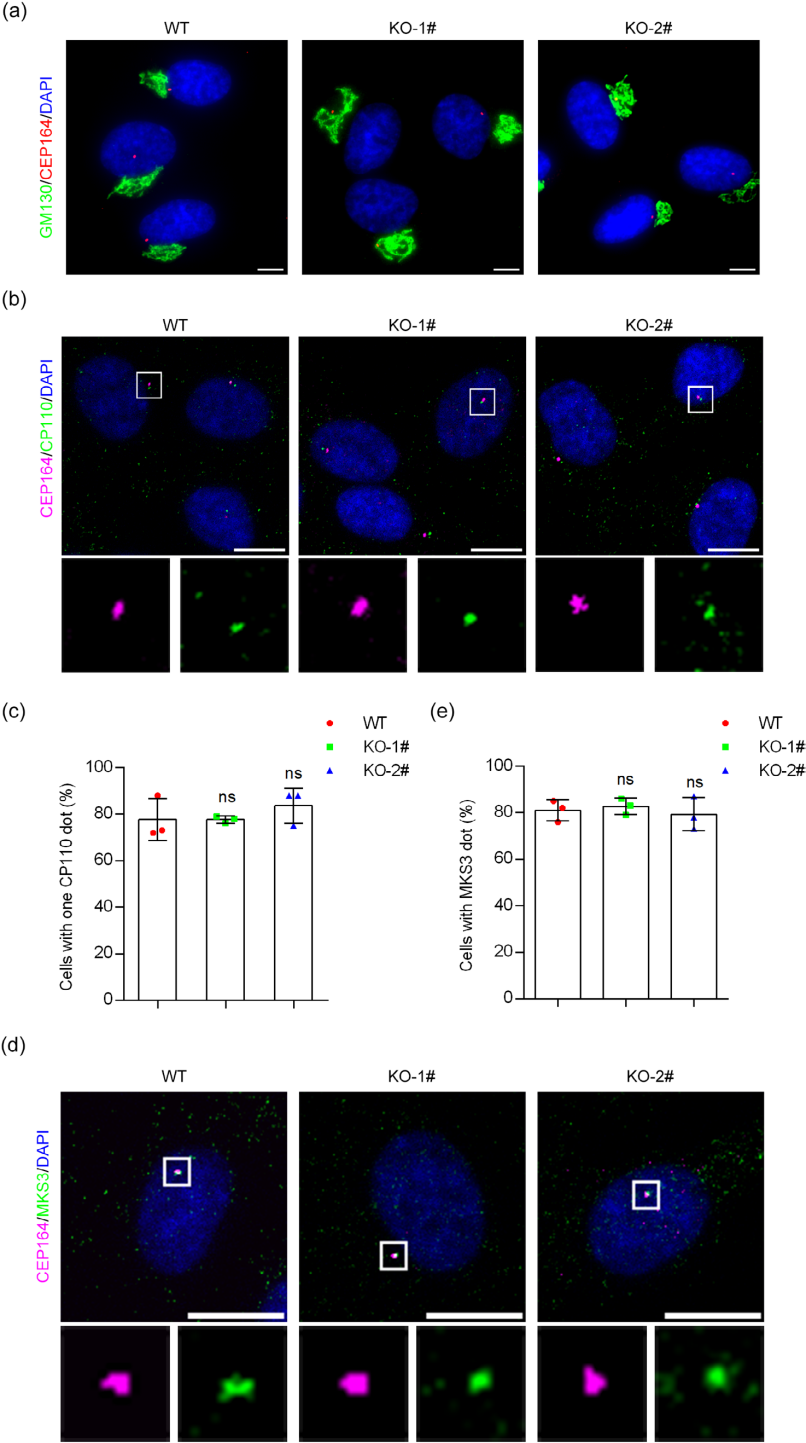
IFT20 is not required for the early steps of ciliogenesis. (a) Immunofluorescence microscopy to detect the Golgi morphology in wild‐type and *IFT20* knockout cell lines (GM130, green; CEP164, red; DAPI, blue. Scale bar, 10 μm). (b) Immunofluorescence microscopy to detect the removal of CP110 in wild‐type and *IFT20* knockout cell lines following serum starvation (CEP164, magenta; CP110, green; DAPI, blue. Scale bar, 10 μm). (c) Quantification of the percentage of cells with one CP110 dot following serum starvation (*n* = 3 independent experiments). (d) Immunofluorescence microscopy to detect the localization of MKS3 in wild‐type and *IFT20* knockout cell lines following serum starvation (CEP164, magenta; MKS3, green; DAPI, blue. Scale bar, 10 μm). (e) Quantification of cells with MKS3 dot following serum starvation (*n* = 3 independent experiments). Data are presented as mean ± SEM. ns, not significant.

In mammalian cells, the formation of cilia involves a series of steps, including the recruitment of pre‐ciliary vesicles to the distal appendage of the mother centriole, formation of ciliary vesicles, removal of CP110 from the mother centriole, recruitment of transition zone components, extension of axonemal microtubules, and formation of the ciliary membrane [38]. As IFT20 depletion decreased the percentage of ciliated cells, we explored whether IFT20 is involved in these processes. Based on the localization of IFT20 at the Golgi apparatus, we speculated that IFT20 depletion might affect the recruitment of vesicles from the Golgi apparatus to the centrosome, which is required for early ciliogenesis. Therefore, we examined the localization of Golgi matrix protein 130 (GM130) by immunofluorescence microscopy. We found that the depletion of IFT20 did not significantly affect GM130 localization (Figure 3a).

CP110 removal from the distal end of the mother centriole is required for basal body formation and precedes axoneme growth. We thus investigated the effect of *IFT20* knockout on CP110 removal. Immunofluorescence microscopy revealed that wild‐type and *IFT20* knockout cells had no significant difference in the percentage of cells with one CP110 dot (Figure 3b,c), indicating that the removal of CP110 was unaffected by the depletion of IFT20. In early ciliogenesis, transition zone proteins are recruited to the mother centriole to form a gate for controlling the material transport into and out of the cilium. Therefore, we examined whether *IFT20* knockout affects transition zone formation. By immunofluorescence microscopy, we found that the localization of Meckel–Gruber syndrome type 3 (MKS3, a marker of transition zone) was unaffected by IFT20 depletion (Figure 3d,e). Taken together, these results suggest that IFT20 is not required for the early steps of ciliogenesis.

### 3.4 Mass Spectrometry Identifies IFT20‐Interacting Proteins

**FIGURE 4:**
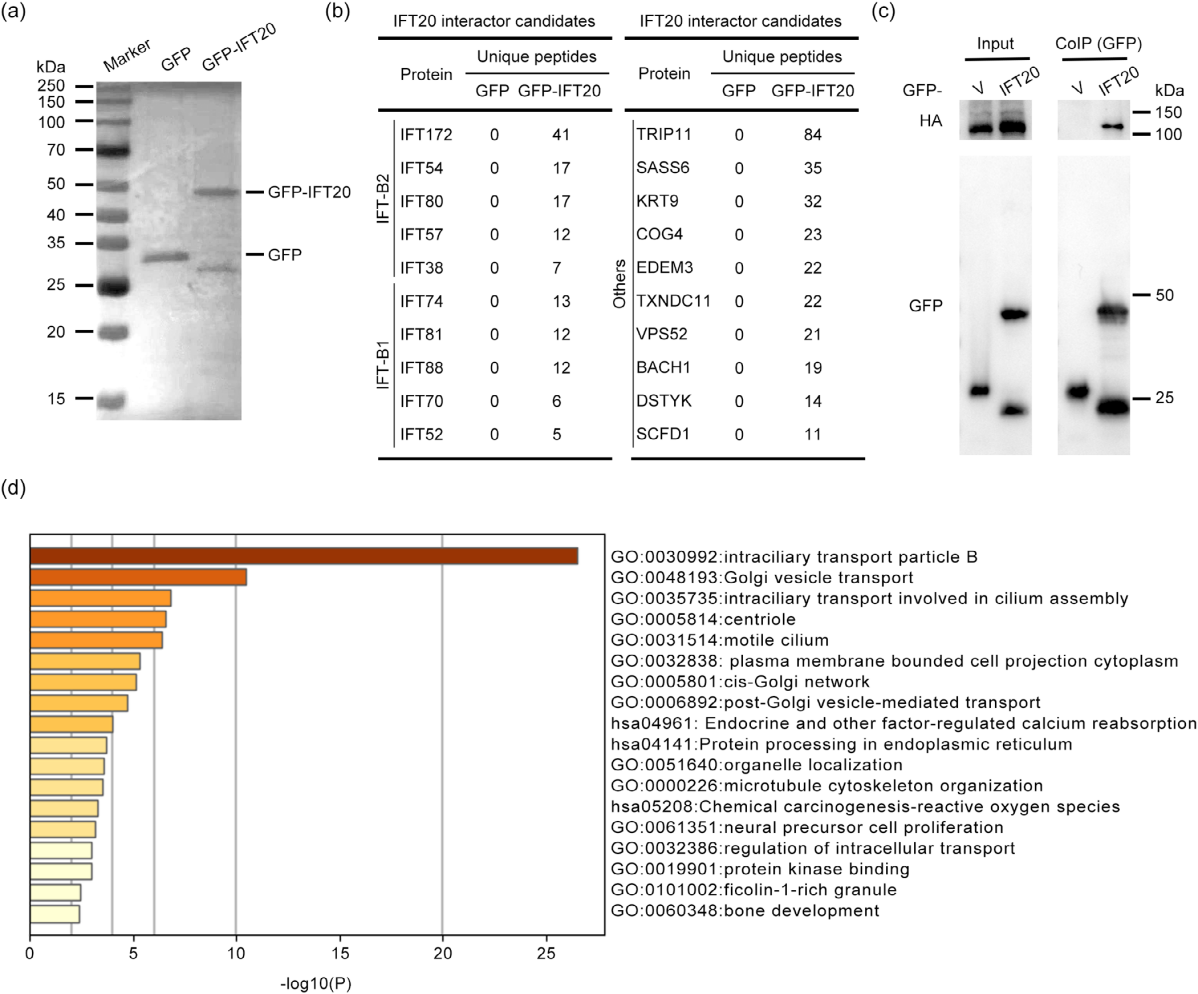
Mass spectrometry identifies IFT20‐interacting proteins. (a) HEK293T cells were transfected with GFP or GFP‐IFT20, and GFP or GFP‐IFT20 proteins were pulled down with GFP magnetic beads and analyzed by mass spectrometry. (b) Proteins that specifically interact with IFT20 were listed based on mass spectrometry data. (c) Immunoprecipitation assay to validate the interaction between exogenous GFP‐IFT20 and HA‐IFT88. (d) Gene ontology analysis generated by Metascape.

To further explore the mechanism underlying IFT20‐mediated regulation of ciliogenesis, we conducted mass spectrometry to identify proteins interacting with IFT20. Human embryonic kidney epithelial (HEK293T) cells expressing GFP or GFP‐tagged IFT20 were lyzed and subjected to immunoprecipitation (IP). The IP samples were verified by immunoblotting (Figure 4a). Mass spectrometry identified a series of proteins of the IFT‐B complex, including IFT88, as candidate IFT20‐interacting proteins. In addition, several proteins not belonging to the IFT complex were identified by mass spectrometry (Figure 4b). The interaction between IFT20 and IFT88 was further confirmed by IP followed by immunoblotting (Figure 4c). Next, we removed nonspecific proteins by comparing hits from GFP and GFP‐IFT20 samples and selected candidate proteins with unique peptides ≥ 5. This analysis ultimately resulted in 51 potential IFT20‐interacting proteins. Gene ontology analysis of these 51 proteins revealed that they are involved in diverse cellular processes, especially IFT and vesicle transport (Figure 4d).

### 3.5 IFT20 Deficiency Decreases the Level and Localization of Other IFT Members

**FIGURE 5:**
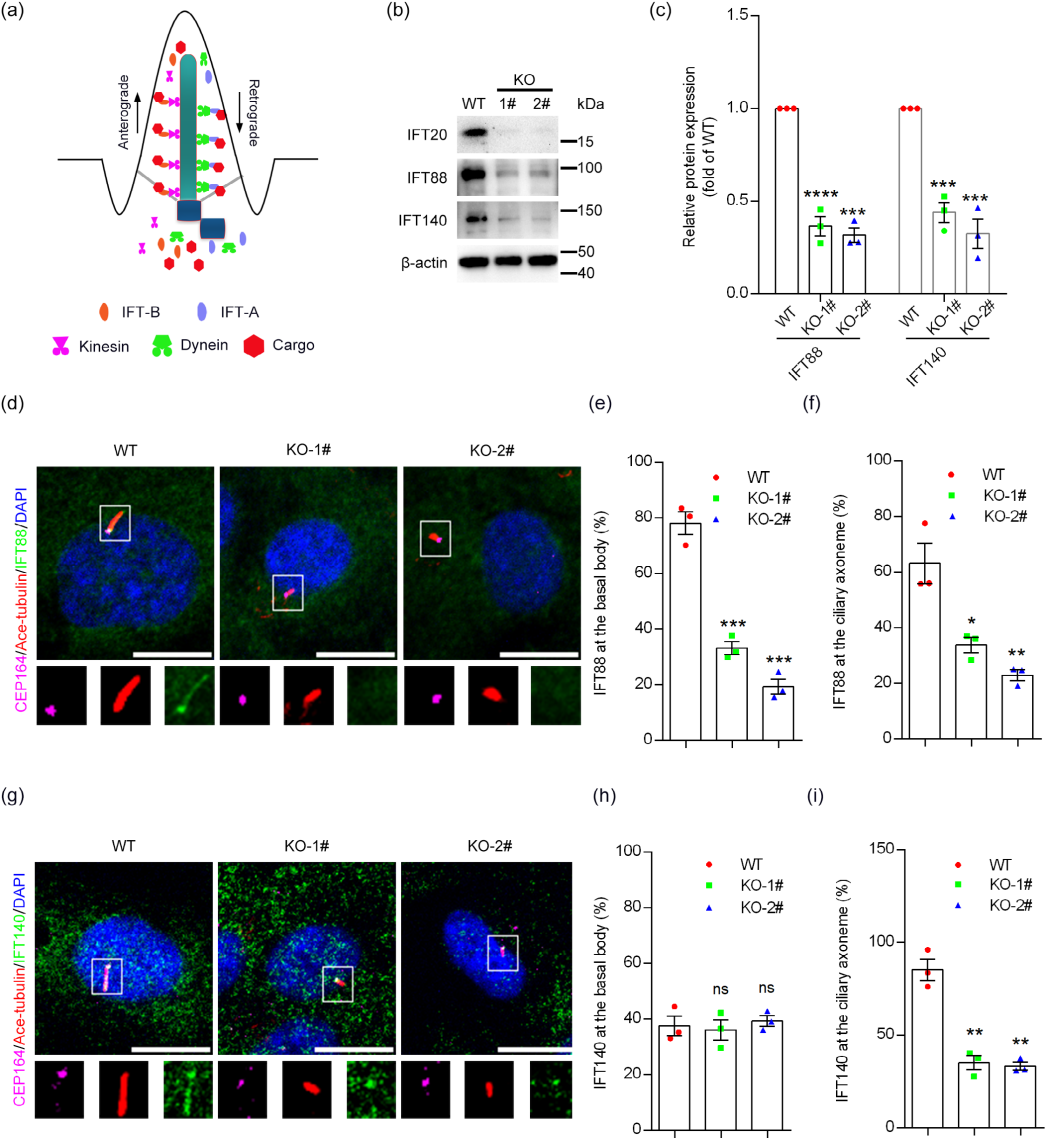
IFT20 deficiency decreases the level and localization of IFT proteins. (a) Schematic representation of IFT‐A and IFT‐B complexes in the cilium. (b) Immunoblotting of IFT88 and IFT140 in wild‐type cells and *IFT20* knockout cell lines. (c) Quantification of levels of IFT88 and IFT140 (*n* = 3 independent experiments). (d) Immunofluorescence microscopy to detect IFT88 in wild‐type and *IFT20* knockout cell lines following serum starvation (CEP164, magenta; Ace‐tubulin, red; IFT88, green; DAPI, blue. Scale bar, 10 μm). (e) Quantification of the percentage of cells with IFT88 at the basal body (*n* = 3 independent experiments). (f) Quantification of the percentage of cells with IFT88 at the ciliary axoneme (*n* = 3 independent experiments). (g) Immunofluorescence microscopy to detect IFT140 in wild‐type and *IFT20* knockout cell lines following serum starvation (CEP164, magenta; Ace‐tubulin, red; IFT140, green; DAPI, blue. Scale bar, 10 μm). (h) Quantification of the percentage of cells with IFT140 at the basal body (*n* = 3 independent experiments). (i) Quantification of the percentage of cells with IFT140 at the ciliary axoneme (*n* = 3 independent experiments). Data are presented as mean ± SEM. *, *p* < 0.05; **, *p* < 0.01; ***, *p* < 0.001; ****, *p* < 0.0001; ns, not significant.

To investigate how IFT20 participates in the IFT process, we analyzed the effect of IFT20 depletion on the levels and localization of IFT‐B and IFT‐A complexes. Specifically, we selected IFT88, a member of the IFT‐B complex, and IFT140, a member of the IFT‐A complex (Figure 5a). We found that the levels of both IFT88 and IFT140 were significantly decreased in *IFT20* knockout cell lines compared to wild‐type cells (Figure 5b,c). Then, we examined the localization of these two IFT proteins by immunofluorescence microscopy. We found that the localization of IFT88 at the basal body and ciliary axoneme disappeared in *IFT20* knockout cells (Figure 5d–f). In contrast, although the localization of IFT140 at the ciliary axoneme disappeared in *IFT20* knockout cells, its localization at the basal body was unaffected (Figure 5g–i). Collectively, these findings suggest that IFT20 orchestrates with other IFT proteins in an exquisite manner to regulate ciliogenesis.

## 4 Discussion

Primary cilia are widely distributed in mammalian cells and are involved in the regulation of many critical physiological processes. Dysregulation of essential ciliary proteins leads to diseases collectively known as ciliopathies [2, 12, 39, 40, 41, 42]. In mouse models, the deletion of IFT20 has been associated with retinal degeneration [31], impaired spermiogenesis and fertility [43], aberrant lymphatic vessel morphology during development and inflammation [44], defective opsin trafficking and photoreceptor outer segment formation [36], and cystic kidney disease [33, 45]. Clinically, mutations or deletions of IFT20 have been linked to ciliopathies affecting the urinary, cardiovascular, skeletal, neurological, immune, reproductive, and respiratory systems [46]. However, the mechanism by which IFT20 regulates ciliogenesis remains unclear. In this study, we generated two *IFT20* knockout cell lines, confirmed the knockout efficiency using immunoblotting and immunofluorescence microscopy, and observed a reduction in both the percentage of ciliated cells and cilium length in these cells.

Ciliogenesis is a multistep process that involves the recruitment of pre‐ciliary vesicles to the distal appendage of the mother centriole [47], formation of ciliary vesicles [48, 49], removal of the CP110‐CEP97 complex from the distal end of the mother centriole [50, 51], recruitment of transition zone components [52, 53], extension of the axoneme [17], and formation of the ciliary membrane [54]. To determine which step was disrupted following *IFT20* knockout, we first examined the early stages of ciliogenesis, focusing on vesicle recruitment to the distal appendages of the mother centriole [55]. As IFT20 uniquely localizes to both the cilium and Golgi apparatus among IFT proteins, we examined whether the Golgi was affected in *IFT20* knockout cells. Our results showed that Golgi morphology, based on the detection of GM130, was not altered, indicating that Golgi function was likely unaffected. However, the status of Golgi‐derived vesicles in ciliogenesis requires further investigation [37].

The removal of the CP110–CEP97 complex from the mother centriole is a key step in ciliogenesis. Failure in this step prevents ciliary axoneme extension even under the stimulation of serum starvation. Therefore, we examined CP110 removal in the *IFT20* knockout cells. We found that CP110 could be removed normally after serum starvation in the *IFT20* knockout cells. Additionally, before axoneme growth, the Y‐shaped transition zone structure connects the ciliary membrane to axoneme microtubules that serve as a gate for the movement of material in and out of the cilium [56]. It has been shown that the deletion of transition zone proteins results in cilium defects. Therefore, we also examined the changes in transition zone assembly in *IFT20* knockout cells. Our findings indicated that the transition zone protein could be recruited normally in the *IFT20* knockout cells. These results suggest that IFT20 is dispensable for basal body transformation during ciliogenesis. To further explore the molecular mechanism of IFT20, we performed mass spectrometry using GFP‐tagged IFT20. Analysis revealed that several IFT proteins interact with IFT20, indicating that IFT20 might affect ciliogenesis by influencing IFT‐mediated substance transport. Gene ontology analysis of mass spectrometry data also discovered many Golgi proteins, suggesting a potential involvement of Golgi vesicle transport in the ciliary function of IFT20.

Our study revealed that ciliary length was reduced in *IFT20* knockout cells, which might be related to the restricted transport processes of the cilium. Therefore, we focused on IFT proteins, which control cargo transport within the cilium [24]. IFT88 and IFT140 were selected as representatives of the IFT‐B and IFT‐A complexes, respectively [57, 58, 59]. We found that after *IFT20* knockout, both IFT88 and IFT140 levels were decreased, and the localization of IFT88 at both the basal body and ciliary axoneme disappeared in *IFT20* knockout cells after serum starvation. In contrast, IFT140 retained the localization at the basal body but lost the ciliary axoneme distribution. As IFT‐B is responsible for anterograde transport required for cilia formation, the impairment of the distribution of IFT‐B proteins at the basal body and ciliary axoneme by IFT20 depletion potentially causes the shortened cilia in *IFT20* knockout cells. However, further research is needed to clarify IFT20's role in the IFT‐B complex, as IFT20 is a peripheral, not a core, component of the IFT‐B complex [60].

## Author Contributions

Q.L., J.Z., and H.Z. supervised the project. W.W. and Y.S. performed the experiments. W.W., Y.S., D.L., and R.L. analyzed the data. W.W., J.Z., H.Z., and Q.L. wrote the manuscript.

## Conflicts of Interest

The authors declare no conflicts of interest.

## Data Availability

All the data that support the findings are presented in the manuscript.
